# Experimental manipulation shows that the white wing patch in collared flycatchers is a male sexual ornament

**DOI:** 10.1002/ece3.48

**Published:** 2011-12

**Authors:** Maaike E de Heij, Lars Gustafsson, Jon E Brommer

**Affiliations:** 1 Bird Ecology Unit, Department of Biological and Environmental Sciences, University of HelsinkiHelsinki, Finland; 2 Animal Ecology, Department of Ecology and Evolution, Uppsala UniversityUppsala, Sweden

**Keywords:** Bird, extra-pair paternity, mate choice, polygeny, sexual selection

## Abstract

Descriptive analysis suggests that a conspicuous white wing patch in dichromatic (black and white) pied and collared flycatchers is under sexual selection. Here, we use an experimental approach to test whether this trait is indeed the target of selection. We caught 100 collared flycatcher *Ficedula albicollis* males soon after their arrival on the breeding site. We reduced (blackened) part of the white wing patch in half of these males and recorded their mating success and within and extra-pair offspring production. Reduction of the size of the white wing patch lowered a male's probability to attract a secondary social female, but not a primary female. However, primary females paired to males with a reduced wing patch were smaller (in tarsus), suggesting that male choice of partner or female–female competition over mates occurs in this species. The probability of pairing with a primary female (but not other components of male reproductive success) declined with arrival time (proxied by the date of capture). Males with a reduced wing patch size tended to sire less extra-pair offspring, although this relationship was reversed in one of the three study plots, suggesting that mating dynamics are context dependent. While our findings show that wing patch size is the target of sexual selection, the pathways and the strength of selection on this ornament differed markedly from a previous descriptive study. Nonexperimental studies of sexual selection in the wild may overestimate its importance because male fitness and ornamentation both depend positively on environmental conditions.

## Introduction

In sexually reproducing species, males often possess extravagant traits, so-called ornaments. These ornaments are thought to result from coevolution of the ornament in one sex (typically the male) and preference for that ornament in the other sex (typically the female) (Andersson 1994; Kirkpatrick and Barton 1997). This coevolution is driven by the superior fitness of individuals with exaggerated ornamentation, either because such ornaments are preferred (Fisherian sexual selection, Fisher 1930) and/or because they are associated with high viability (Zahavi 1975). Nevertheless, demonstrating that a conspicuous element of a male's appearance is indeed under sexual selection is challenging, especially under natural conditions. Two approaches are particularly attractive in wild avian populations. First, the ornament may be experimentally manipulated before the reproductive season in order to study its consequences (e.g., Sheldon et al. 1999). This procedure explicitly explores whether a specific ornament is the target of sexual selection. Second, many bird species have a high proportion of extra-pair young (EPY), which are fathered by males that do not contribute care. EPY presumably result from differential extra-pair mating success across males, possibly according to their external attributes (Griffith et al. 2002, but see Arnqvist and Kirkpatrick 2006). Hence, an association between a male ornament and the production of EPY is considered indicative of sexual selection in progress.

One of the best studied wild systems in which male ornamentation and female mate choice have been investigated is the collared flycatcher *Ficedula albicollis*, a small, migratory passerine. The species has a moderate rate of extra-pair paternity varying from 16% to 21% of all nestlings (based on three populations, Brommer et al. 2010). In addition, bigamy is fairly common since 9–14% of all nests are broods where the social father also has a primary nest with another female (Gustafsson and Qvarnström 2006). Hence, there is a reasonable scope for sexual selection in this species. Males are dichromatic (black and white) with conspicuous white patches on forehead and wings. Most studies have focused their attention on the importance of the forehead patch in mate selection. The size of the forehead patch is heritable (Garant et al. 2004; Qvarnström et al. 2006), and condition dependent (Gustafsson et al. 1995; Garant et al. 2004). Male ornament and male annual fitness are positively correlated on the genetic level indicating that genes for a large forehead patch also code for high fitness (Qvarnström et al. 2006). The forehead patch is thought to be important for male–male competition (Qvarnström 1997; Qvarnström et al. 2000a) and sperm competition (Sheldon et al. 1997; Sheldon and Ellegren 1999).

In addition to forehead patch, Sheldon and Ellegren (1999) found that the size of the white patch on the wing (measured on the primaries) plays a role in sexual selection—in particular in sperm competition (limiting extra-pair paternity in a male's own nest and gaining it in other males’ nests). However, this ornament has received much less attention compared to the forehead patch. Yet, males clearly display their wing patches during courtship behavior, both during male–male competition (Török et al. 2003) and courtship flights. Garant et al. (2004) shows that the size of the forehead patch and that of the wing patch are weakly genetically correlated (genetic correlation of 0.12 ± 0.02 [SE] in adults). In a Hungarian population of collared flycatchers, Török et al. (2003) show that the white wing patch size in males is heritable and condition dependent, which both are necessary properties for an informative signal. In the pied flycatcher *F. hypoleuca*, a closely related species, the probability of being cuckolded is negatively related with the ultraviolet (UV) chroma of the white wing patch in males (Lehtonen et al. 2009). These findings suggest that the wing patch could be an important ornament in sexual selection. However, there is—to our knowledge—only descriptive and no experimental evidence linking variation in the size of the white wing patch to differential male mating success. Condition dependence (sensu Price et al. 1988) can generate a spurious phenotypic correlation between an ornament and male mating success, in case both aspects are positively influenced by an underlying environmental variable (“condition”). Only experimental manipulation of the size of an ornament will allow to critically assess its role in driving a male's mating success.

In this study, we carry out a manipulation of the size of the white patches on the wing of male collared flycatchers in order to study their importance in sexual selection. Collared flycatcher males defend a territory after arriving from overwintering in Africa. Females presumably arrive later, rapidly choose their partner and start to breed (Lundberg and Alatalo 1992). We caught males early in the breeding season, soon after arrival, and reduced the amount of white on their wing patches. During the breeding season, we monitored the nest boxes to follow breeding attempts and to identify the parents. We used molecular methods to quantify within and extra-pair offspring production. We hypothesize that if the wing patch is a sexual ornament that females use to select their mates, males with reduced wing patches should have a lower breeding success compared to nonmanipulated males, either because of a lower probability to get paired, or because of a reduction in the offspring sired in either their own nest or the nest of other females.

## Material and Methods

### Study population

We conducted the experiment in 2008 in a nest box breeding population of collared flycatchers on the southern part of the Swedish island of Gotland (57°30′N, 18°30′E). Our study area consisted of three different nest box areas in the core study area of a long-term study on this species carried out in southern Gotland since 1980. Our study area consisted of the nest box plots “Anderse” (61 boxes), “Fide Prästäng” (159 boxes), and “Tuviken” (204 boxes). Both Anderse and Fide Prästäng are deciduous forests dominated by Pedunculate Oak *Quercus robur* and Ash *Fraxinus excelsior*. Tuviken is a mixed coniferous forest dominated by pine *Pinus silvestris* and birch *Betula pubescens*.

### Experimental procedure

Male collared flycatchers arrive at the breeding grounds early in spring. Newly arrived males frequently visit nest boxes to establish a breeding territory. In the year of this experiment, the first male flycatcher was spotted in southern Gotland on the 24th of April in an area neighboring Fide Prästäng. From 26 April to 21 May, we caught males as close upon their arrival at the breeding grounds as possible. Males were caught using traps inside the nest boxes, which were triggered when males entered nest boxes. We placed traps either in all empty nest boxes in our experimental study plots, especially focusing on those nest boxes where males were actively singing. Nest boxes that were already taken by flycatchers or other bird species were not included. In each experimental area, we made a catching attempt every second day early in the morning (from 5:30 until 12 o'clock).

We measured the biometrics of each male following prior developed protocol. Tarsus length (in mm), size of the forehead patch (height times width in mm), and size of the wing patch (sum of the amount of white on primaries 3 till 7, in mm) were measured using a sliding calliper. Individuals were weighed (in g) using a spring balance. We also took a blood sample from the brachial vein of one of the wings. The blood was stored in 96% ethanol and kept for parentage analyses. Most (95%) of the measurements and all the painting of the males were performed by two people. Any unringed male was ringed to allow lifelong identification.

After measuring them, male collared flycatchers were random systematically assigned to treatment groups within the experiment by tossing a coin at the start of every catching day and then alternating treatments throughout that catching day. The measurement of “wing patch” in published literature on this population (e.g., Sheldon and Ellegren 1999; Garant et al. 2004) specifically refers to the amount of white on the outer primaries measured as described above. However, collared flycatchers have a band of white (which is particularly large in males) that extends almost over the full length of the wing, including the secondary wing feathers and the tertials. For the males in the “reduced” treatment group, we reduced the amount of white on the primaries, secondaries, and outermost three tertials to a narrow band by blackening part of the white wing patch with a marker pen (COPIC 110 special black). We gave males in the “control” group the same treatment as the “reduced” males, but used a marker pen without ink (COPIC 0 colorless blender). These procedures are illustrated in the Supporting information (Figs. S1 and S2). The painting of the feathers did not influence the reflectance of UV wavelengths (Veen et al. 2009). Males in this population are all clearly black and white (not brownish and white). The blackening by the marker pen approximately matched the black of the plumage (Figs. S1 and S2 show typical male coloration). Although the color faded by the time a painted male was recaptured feeding nestlings to brownish black it did not turn “dirty white.” We hence assume that males with an experimentally blackened wing patch had the appearance of a male with a small wing patch size throughout the period of mate acquisition. After the manipulation, each male was released close to the nest box where he was caught. From here onwards, we refer to the group of males whose wing patch was reduced or control treated as “experimental males.”

### Data collection

From the first catching day onwards, we daily checked all nest boxes in our study area to record the onset of nest building. We used the onset of nest building as the moment of pair formation. A collared flycatcher nest typically consists mainly of dead leaves from the previous year that are picked up from the litter layer. Such leaves are abundant on the forest floor and nest building typically proceeds fast (few days). Because detecting female collared flycatchers in the foliage is difficult, we could not reliably establish pair formation by direct observation.

After we established the onset of nest building, we checked these boxes every third day for laying date and clutch size. From the expected hatch date onwards, we checked daily whether the nest would hatch. On day 2 (hatching is day 0), we collected a small blood sample from chicks from the femoral vein for parentage analysis and clipped their nails in a unique combination to allow individual identification. At the same time, we collected unhatched eggs or dead chicks for parentage analyses. We ringed nestlings at day 8 when their nail clippings were still clearly visible, and measured them again at day 12 (close to fledging).

We caught females during incubation, and males when feeding nestlings. All individuals were identified individually by their ring or were ringed if they did not have a ring. Biometric measurements were taken and a blood sample was collected from the brachial vein.

### Paternity analysis

All laboratory work and parentage analyses were performed by the Center of Evolutionary Applications (University of Turku, Finland). DNA was extracted with salt extraction method modified from Aljanabi and Martinez (1997). Samples were genotyped with 12 markers divided into two panels (panel 1 Fhy370, Fhy453, Fhy466, Fhy450 and panel 2 Fhy401, Fhy408, Fhy304, FhU2, Fhy328, Fhy339, Fhy228, Fhy448; Leder et al. 2008). The marker FhU2 corresponds to PTC2 in Ellegren (1992). For each panel, the PCR was carried out in one 8 µl multiplex using QIAGEN Multiplex PCR Kit (Qiagen Inc. Valencia, CA) with the annealing temperatures of 55°C and 56°C for panels 1 and 2, respectively, and the primer concentration varying from 0.05 to 1.2 µM. Otherwise, the PCR amplification and electrophoresis were performed as outlined in Lehtonen et al. (2009). A single PCR with the annealing temperature at 55°C was performed using QIAGEN Multiplex PCR Kit and the subsequent fragments were visualized by capillary electrophoresis together with microsatellite panel 1.

The paternities were determined by exclusion in all individuals that had been genotyped at four or more loci. A single nonexcluded candidate father could be determined for 96.5% of the offspring (*N* = 612; of which 412 chicks belonged to nests with an experimental social father), 21 individuals had more than one father candidate, and five individuals did not have sufficient genotype data. The program Cervus 3.0 was used to facilitate the exclusion process and to calculate the nonexclusion probabilities (Kalinowski et al. 2007). The genotypes were rechecked for errors or possible mutations in the cases when one mismatch occurred between the individual and the most likely father (*N* = 19). Nestlings with matching genotypes with both the social male and an extra-pair male were always assigned to the social male (*N* = 10). Similarly, in cases where the clutch in one nest box had mixed fathers and if some of the offspring matched two candidate males, the paternity was assigned so that the total number of fathers per clutch was minimized (*N* = 11).

### Criteria for inclusion in the analysis

In total, we manipulated 100 males of which 50 males had their wing patch reduced and 50 males were treated as control. An experimental male was included in the analysis in case his wing patch was either reduced or control painted before attracting a mate (as judged by the onset of nest building as described above). We could establish the mating and reproductive success of these experimental males by catching them at the nest box while feeding nestlings. We assumed that all the males that we trapped inside the nest box were the social fathers of that nest. In case males were not caught at a nest box (mostly because they did not feed the chicks or the nest was abandoned before we could try to catch the males), we could retrace them based on parentage analyses of the blood samples taken at the first catch. When >80% of the chicks in one nest belonged to one male, we assigned that male to be the (social) father of the nest.

Some males turned out to be polygynous; they were found to be the social father at two nests with different females. We refer to the nest and female with the earlier laying date as “primary,” and the later one as “secondary.”

### Statistical analysis

The reproductive success of the males was the focus of our analyses. We analyzed three aspects of male success relating to (1) mating and reproductive success with a primary female, (2) mating and reproductive success with a secondary social female, and (3) success in gaining extra-pair paternity. Besides treatment (wing patch reduced or control painted), we included date of capture and the area in which a male was caught as independent variables in order to control for variation in these variables across males that our experimental setup could not standardize for. Because treatment was randomized, we do not include any male characteristics in our analysis. When analyzing biometrics, we included the identity of the observer as a fixed effect in order to control for a possible observer effect on measurements.

### Primary nest

We analyzed the probability to get a primary female, and the probability of getting cuckolded using a binary logistic regression, and the probability of siring young in its own nest (number of sired young/total number of young) using a Generalized Linear Model (binomial error distribution and logit link function). We analyzed the characteristics of the primary females that were attracted by the experimental males using a General Linear Model. We contrasted the size of their wing patch and their tarsus, and their weight during incubation (one missing value) between control and reduced painted males.

### Secondary nest

A certain proportion of collared flycatcher males mate, in addition to their primary female, also with a second social female. Males attend these secondary nests at a low rate. We analyzed the probability to get a secondary social female using a binary logistic regression. The sample size included all the experimental males that were recaptured in the study area and that were manipulated before they obtained a secondary social female. We analyzed the characteristics of the secondary social females attracted by comparing the characteristics (wing patch, tarsus, and weight at incubation) of the secondary social female with that of the primary female in a paired *t*-test, based on the measurements corrected for observer effect.

### EPY in other nests

Collared flycatcher broods may include offspring that have been sired by a male that is not the social father, indicating that the female has engaged in extra-pair mating. We analyzed the probability for an experimental male to gain paternity with such an extra-pair female using a binary logistic regression. The sample size included all the experimental males that were recaptured in the study area as social males and some floaters. We analyzed the effect of treatment on the number of EPY sired in other nests using a Generalized Linear Model (poisson error distribution and log link function).

### Male share of paternity in total

We analyzed the male share in paternity for the total number of young sired (i.e., young sired in their own nest—primary and secondary—and in other nests) for all the males (with and without nests) that we recaptured in our area. The total number of young sired was analyzed using a General Linear Model assuming Poisson errors and a log link.

We tested for all two-way interactions, but eliminated them from the basic model using backwards elimination when there was no effect of the interaction. Two-way interactions were only mentioned when statistically significant. We performed all statistical analyses in SPSS 15.0 for Windows.

## Results

### Primary nest

Prior to the experiment, males in the two treatment groups did not differ from each other in the size of their wing patch (GLM; *F*_1,96_ = 0.126, *P* = 0.72, corrected for observer [*n* = 3]) or their forehead patch (GLM; *F*_1,96_ = 1.477, *P* = 0.23, corrected for observer [*n* = 3]).

**Figure 1:**
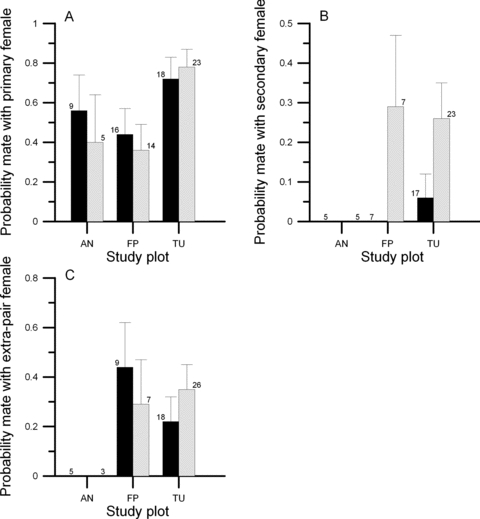
Pairing probability for experimental males in relation to the study plot they were caught in, and the manipulation (reduced or control) of their wing patch size. For each study plot (for acronym, see Table 1), the mean probability with standard error for control painted males are plotted in light gray, and for reduced painted males in black. Plotted are (A) the probability to pair with a primary female (*n* = 85 males; Table 2A), (B) to pair with a secondary social female conditional upon having a primary female (*n* = 62 males; Table 3), and (C) to mate with an extra-pair female (*n* = 68 males; Table 4A). Sample sizes broken down in the different categories are indicated above the bar.

Of the 100 males that we experimentally manipulated early in the season, 66 males (34 control, 32 reduced) were found to attend a nest later in the season (Table 1). Of these 66 males, we had to discard 15 males for the analysis of mating and reproductive success with a primary female. This was either because the manipulation was done after pair formation (*n* = 11), or because they were recaptured outside our study area and subsequently we did not have information about the timing of pair formation (*n* = 4). The probability to pair with a primary female was high early in the season and declined as the season advanced (Table 2A). The probability of getting paired within the study area differed between the areas in which we performed the experiment; males in the area Tuviken were more likely to get paired than in the other two areas. The probability to get paired, however, did not depend on the experimental treatment (Table 2A, Fig. 1A). Because the wing patch reduction could have a differential effect in older males, we additionally tested for a “treatment × age (yearling vs. adult)” interaction on the probability to obtain a primary female, but this was not significant (Table S1).

**Table 1:** Overview per area and per treatment of the number of males caught and manipulated early in the season, the number recaptured while breeding and the number of males we manipulated before pair formation with the primary female (“manipulated prior to pairing”). In the analysis of pair formation with a secondary social female, all 62 males recaptured in the three study plots could be included. In addition, four individuals were recaptured outside the study plots (termed here “emigration”)

Area	Treatment	Manipulated	Recaptured	Manipulated prior to pairing
Anderse (AN)	Control	6	3	2
	Reduced	10	6	6
Fide Prästäng (FP)	Control	16	7	6
	Reduced	17	6	6
Tuviken (TU)	Control	28	23	18
	Reduced	23	17	13
All	Control	50	34	26
	Reduced	50	32	25
Total		100	62	51
Emigration	Control		1	
	Reduced		3	

**Table 2:** Model results for the effect of treatment, area, and date of capture on (A) the probability to get a primary female, (B) the probability of getting cuckolded, and (C) the male share of paternity in its own nest. When significant, the interaction is reported. When there were significant differences between the areas, the contrast of the individual areas (in relation to the area “Tuviken”) is reported. Significant (*P* < 0.05) variables are printed in bold

Term (Level)	Coefficient	Wald χ^2^	df	*P*
A. Probability to pair with a primary female				
**Intercept**	2.36 ± 0.66	12.8	1	<0.001
Treatment (Reduced)	0.099 ± 0.50	0.04	1	0.84
**Area**		12.9	2	0.002
Anderse	−1.62 ± 0.74			
Fide Prästäng	−2.24 ± 0.63			
Date of capture	−0.120 ± 0.041	8.8	1	0.003
B. Probability to be cuckolded				
Intercept	−0.51 ± 0.58	0.8	1	0.38
Treatment (Reduced)	−0.022 ± 0.60	0.001	1	0.97
Area		0.6	2	0.75
**Date of capture**	0.007 ± 0.050	0.021	1	0.89
C. Male share of paternity in own nest				
**Intercept**	1.77 ± 0.41	40.9	1	<0.001
Treatment (Reduced)	0.53 ± 0.35	2.3	1	0.13
Area		4.6	2	0.10
Anderse	2.15 ± 1.01			
Fide Prästäng	0.37 ± 0.50			
Date of capture	0.010 ± 0.033	0.84	1	0.36
**Area × Date of capture**		9.9	2	0.007
Anderse × Date	−0.29 ± 0.11			
Fide Prästäng × Date	0.131 ± 0.096			

The females that paired with the males of the two treatment groups differed in tarsus length (treatment, *F*_1,48_ = 7.57, coefficient –0.33 ± 0.12, *P* = 0.01). Females that were mated with males of the control group had larger tarsi than those that were mated with males from the reduced group. They did not differ in the size of their wing patches (treatment, *F*_1,48_ = 1.493, *P* = 0.23) or in the weight during incubation (treatment, *F*_1,47_ = 0.56, *P* = 0.46).

In their primary nests, males had on average 5.75 ± 1.32 (SD) young in their nest, of which they sired on average 4.86 ± 1.77. The probability of getting cuckolded did not differ between the two treatment groups, and did not depend on any of the other explanatory variables tested (Table 2B). The probability that an experimental male sired young in its own nest did not depend on the experimental treatment (Table 2C), but did depend on the date of capture, although this latter effect differed between the areas. In the area Anderse, the probability of siring young in its own nest decreased strongly with date of capture, while in the other two areas the probability did not depend on date of capture.

### Secondary nest

We could include 62 experimental males that were recaptured breeding in our study area for analysis of mating and reproductive success with a secondary social female (Table 1). Of these 62 recaptured males, nine males (eight control, one reduced) were bigamous. Of these nine males, seven males were breeding in the area “Tuviken” and two in the area “Fide Prästäng.” The probability that a male obtained a second partner after they were already successfully paired depended on the experimental treatment (Table 3, Fig. 1B). Males that retained their original size of their wing patch were more likely to attract a second female than the ones that got the size of their wing patch reduced. Other explanatory variables tested did not explain any variation (Table 3).

**Table 3:** Model results for the effect of treatment, area, and date of capture on the probability to get a secondary social female

	df	Wald χ^2^	Coefficient ± SE	*P*
Intercept	1	2.213	−1.041 ± 0.700	0.14
Treatment	1	3.698	−2.122 ± 1.104	0.05
Area	2	0.011		1.00
Anderse	1	0.000	−19.289 ± 13348	1.00
Fide Prästang	1	0.011	−0.099 ± 0.950	0.92
Date of capture	1	0.013	0.006 ± 0.057	0.91

The females that got attracted as secondary social females had smaller tarsi than the ones that got attracted as primary females (paired *t*-test; *t* = 2.365, df = 8, *P* = 0.05). The females did not differ in wing patch size (paired *t*-test; *t* = 1.049, df = 8, *P* = 0.33) or weight during incubation (paired *t*-test; *t* = 0.228, df = 8, *P* = 0.83).

In their secondary nests, males had on average 5.56 ± 1.13 (SD) young in their nest, of which they sired on average 4.00 ± 1.94.

### EPY in other nests

Over all the nests in the study area, there were 634 offspring sampled and 19.4% of them (*n* = 123) were sired by another male than the social father. In the subset of experimental males that were manipulated prior to pairing, 47 young were sired by 19 experimental males. Interestingly, six of these were males not identified as having an own nest (“floaters” or males whose nest was deserted before catching or who nested in a natural cavity).

In total, 11 males belonged to the control painted group and eight males belonged to the reduced painted group. Males of the two treatment groups did not differ in the probability to obtain an extra-pair female (Table 4A). The number of extra-pair young depended on treatment, but the effect differed between the areas (Table 4B, Fig. 1C). In Fide Prästäng, the number of extra-pair young gained was higher for males of the reduced treatment, while in Tuviken the effect was the opposite. The number of young sired also depended on the date of capture, but the effect differed between the areas (Table 4B). In the area Fide Prästäng, the number of extra-pair young gained increased with date of capture, while in Tuviken the number of young sired did not depend on date of capture.

**Table 4:** Model results for the effect of treatment, area, and date of capture on (A) the probability to get an extra-pair female and (B) the number of extra-pair young gained

Term (Level)	Coefficient	Wald χ^2^	df	*P*
A. Probability to get an extra-pair female				
Intercept	−0.60 ± 0.56	1.2	1	0.28
Treatment (Reduced)	−0.21 ± 0.57	0.1	1	0.71
Area		0.2	2	0.91
Date of capture	−0.019 ± 0.041	0.2	1	0.65
B. Number of extra-pair offspring produced				
Intercept	0.30 ± 0.30	1.5	1	0.22
Treatment (Reduced)	−0.16 ± 0.38	3.4	1	0.07
**Area**		4.1	1	0.04
Fide Prästäng	−2.04 ± 0.82			
**Treatment × Area**		4.9	1	0.03
Reduced × Fide Prästäng	1.87 ± 0.85	4.9	1	0.03
Date of capture	−0.069 ± 0.030	0.001	1	0.97
**Area × Date of capture**		10.9	1	<0.001
Fide Prästäng × Date	0.14 ± 0.042			

### Strength of sexual selection on male wing patch size

In order to obtain an integral measure of male fitness, we can consider the sum of offspring males sired in primary and secondary broods. Because the manipulation of wing patch size did not affect mating success with primary females, we also include the six individuals that were manipulated after pair formation with their primary female in order to obtain a more complete measure. The total number of offspring sired by an experimental male (*n* = 68) during the entire breeding season was on average 5.82 ± 3.16 (SD). The number of offspring sired did not depend on the experimental treatment (mean ± SE—control (*n* = 36): 5.89 ± 0.55; reduced (*n* = 32): 5.75 ± 0.54; *t*_66_ = –0.180, *P* = 0.86). Hence, the experimental “removal” of the wing patch has an effect size of 2.4% ([5.89–5.75]/5.89) reduction in male reproductive fitness.

## Discussion

Collared flycatchers have a conspicuous white wing patch, which is thought to act as an ornament in sexual selection (Sheldon and Ellegren 1999). Here, we compare the pairing and reproductive success of males that had their wing patch reduced versus those that were control painted before the onset of nest building (taken as a proxy for pair formation). The clearest support for the wing patch as a male sexual ornament is that experimental reduction of the wing patch lowers the probability to attract a secondary social female. Although pairing success with primary females did not differ between the experimental groups, males with a reduced wing patch paired with smaller females than control painted males. Lastly, in terms of the production of EPY, the consequences varied in that the experimental reduction of wing patches lowers the number of EPY sired in one area, but not in the other.

Polygeny is an important feature of collared flycatcher life history. Approximately 9–14% of all nests are a secondary brood (Gustafsson and Qvarnström 2006), but it is often difficult to reliably detect these, because males provide far less care for their secondary social female than their primary female (Qvarnström et al. 2003) that hampers trapping them at their secondary nest (which is needed for identification). In our study, we used, apart from capture data, also molecular paternity analysis, which allowed us to identify more than half of the bigamous males (five out of nine males), without physically trapping these males at the nest. In total, 14.6% (9/62) of males that had a primary female and 9% (9/100) of all experimental males caught before the breeding season obtained a secondary social female, indicating that bigamy presents a considerable fitness potential for an individual male, although it should be noted that the success rate of these nests usually is low (Qvarnström et al. 2003).

We find experimental evidence that secondary social females prefer to mate with males with large wing patches. Females may base this choice on the potential direct benefits associated with males with a large ornament. Evidence for such direct benefits associated with ornaments in the male collared flycatcher mainly stems from work on the forehead patch. Secondary nests are—by definition—later than primary nests, and females are presumably not aware whether they are primary or secondary (Alatalo et al. 1987). Females that breed late in the season are thought to make an adaptive choice for a male with a large forehead patch, because broods sired by such males are more productive compared to broods of females partnered with a male with a small forehead patch (Qvarnström et al. 2000b). Hence, secondary social females may show a stronger preference of ornamented males than primary females resulting in a lower probability for acquiring a secondary social female for males with a reduced wing patch size. On the other hand, the experimental reduction in male wing patch size did not have a stronger effect in late-breeding primary females (i.e., the “treatment—date of manipulation” interaction dropped from the model), suggesting that seasonal timing per se was not the driving factor. Another possibility is that males with a reduced wing patch are hampered in obtaining a good-quality second territory that is needed to attract a secondary social female. Collared flycatcher ornaments play a role in male–male competition, although most work to date has considered the forehead patch (Qvarnström et al. 2000a). Males with a reduced wing patch may be exposed to more aggressive interactions and may have difficulties in occupying two territories.

A second main finding is that although the rate of pairing with primary females is not affected by wing patch size, experimental reduction in male wing patches results in pair formation with smaller females. In general, female size is (weakly) positively related to fitness in this population (Merilä and Sheldon 2000; J.E. Brommer and L. Gustafsson unpubl. data: females with one SD larger tarsus enjoy 3.5% increase in lifetime fitness). Further evidence indicating that smaller females are of lower quality comes from our finding that secondary social females are smaller than primary females. The traditional view of mate selection in flycatchers is that males arrive early in order to obtain and defend high-quality territories, and females choose the territory (Alatalo et al. 1986). The observed size difference in the primary females of the reduced versus the control painted males therefore indicates either an element of male choice (for a high-quality female) or of female–female competition over males or the territory they manage to obtain.

One important variable that diminished the probability for a male to pair with a primary female is his date of capture. This decline was not driven by later-caught males not getting a partner because they were already paired, because we (retrospectively) omitted such males on the basis of nest-building activity. Further, after release the within-season emigration from our study plots to surrounding plots presumably was low (we detected the movement of 4/100 males to another plot). Trapping intensity was high. Hence, we believe that most males that were not recaptured did not breed, and that the date of capture is a close correlate of the arrival time of the male in the study area. Thus, this finding indicates that male early arrival is under directional fecundity selection, as expected on the basis of theoretical work (Morbey and Ydenberg 2001; Kokko et al. 2006). Interestingly, the date of capture did not explain variation in any of the other parameters such as obtaining a secondary social female or gain or loss of paternity, although late arriving males gained more extra-pair offspring in one of the two study plots. Thus, selection for early male arrival primarily acts through the probability to pair with their social partner. This finding is in contrast to the expectation that early arrival is beneficial to males through greater opportunity for sperm competition (Birkhead and Møller 1998), for which there is some comparative evidence in migratory birds (Rubolini et al. 2004; Coppack et al. 2006).

A third main conclusion is that there is weak evidence (*P* = 0.07) that males with large wing patches produce more extra-pair offspring. An association between wing patch size and number of EPY sired was expected on the basis of prior descriptive selection analyses. Intriguingly, the effect of the experimental reduction was dependent on the study plot, and actually increased the propensity to sire EPY in one of the study plots. Hence, we find that gaining and loosing paternity was dependent on the context (i.e., the study plot). Because replication is lacking, we can here only speculate on the potential causes. One possibility is that reduction of wing patch size makes a male more cryptic, which could aid in obtaining extra-pair matings when density is high or when the vegetation is dense, both of which apply to the plot “Fide Prästäng” where the reduction of the wing patch led to an increase in the number of EPY. Similarly, males with a large forehead patch are relatively less successful in gaining EPY in plots with high compared to low population density (Hjernquist 2008). Such context dependency in the production of EPY may provide insights into which factors drive extra-pair mating dynamics and thus sexual selection (Griffith et al. 2002). Manipulation of male ornaments in a migratory bird prior to pairing across a wider (and possibly controlled) range of environments may be a way forward to obtain further insight into this issue in the wild.

The weak effect of the reduction in wing patch size on the probability to gain extra-pair offspring was consistent with descriptive analyses. However, Sheldon and Ellegren (1999) further demonstrated that in broods with multiple sires, extra-pair paternity is gained by a male with a larger ornament than the social male (i.e., relative ornament size matters). If this pattern was because of females preferentially mating with males with large ornaments then we would expect experimental reduction in the wing patch to increase the probability that a male was cuckolded. However, our findings do not support this prediction at all (Table 2B, treatment: *P* = 0.97).

## Conclusions

Our experimental manipulation of wing patch size in males provides evidence that this ornament is a target of sexual selection. The main pathways through which sexual selection appears to act are that a male with a large white wing patch (1) pairs with a larger primary female (i.e., potentially a higher quality female), (2) is more likely to obtain a secondary social female, (3) tends to sire more EPY, although this last aspect appears to depend on the local environment. Taken together, however, we do not find strong sexual selection on this ornament, as the total number of offspring sired for a male whose wing patch size was reduced is only 2.4% lower than for a control painted male. The limited effect of wing patch size reduction on average male reproductive is because this manipulation does not affect a male's probability to obtain a primary mate. A primary female apparently bases her choice of partner on other aspects. Although wing patch size reduction lowers the probability to obtain a secondary social female, this has little consequences for male average fitness, because few polygamous matings occur. Nevertheless, sample sizes in this study were modest and there was considerable uncertainty around the estimates of male reproductive success in the two treatment groups.

Our findings of a low overall impact of wing patch size on reproductive fitness contrast with analysis on the total number of fledglings sired, which suggests that larger wing patch size increases male reproductive fitness (Sheldon and Ellegren 1999). One potential reason for the discrepancy is that we have carried out only a downward manipulation of the ornament. If fitness is accrued exclusively by few males with very large ornaments, then upward manipulation of male's wing patch size may lead to a different conclusion. However, mating in the collared flycatcher is probably not exclusive enough for this explanation to hold, because 64 of the 100 males caught prior to breeding indeed obtained a primary partner. Because the expression of sexual ornaments is dependent on environmental conditions (see e.g., Garant et al. 2004 for collared flycatchers), it is likely that phenotypic selection analysis is inflated by environmental covariance between the ornament and fitness (sensu Price et al. 1988). That is, individuals in good condition display a large ornament and their good condition (rather than their large ornament per se) makes that they also have high fitness. Experimental manipulation avoids such environmental covariance, because only the ornament is manipulated. Our work suggests that descriptive analysis of sexual selection in the wild may overestimate its importance. A comparison of experimental and descriptive studies is therefore needed to improve our understanding of the strength of sexual selection in the wild.

## Supporting Information

**Table S1.** Model results for the effect of treatment, area, date of capture, and age (including interaction of age with treatment) on the probability to get a primary female.

**Figure S1.** Pictures of the wing patch prior (A) and after (B) experimental reduction.

**Figure S2.** Pictures of the wing patch prior (A) and after (B) control painting.

## References

[b1] Alatalo RV, Lundberg A, Glynn C (1986). Female pied flycatchers choose territory quality and not male characteristics. Nature.

[b2] Alatalo RV, Götlander K, Lundberg A (1987). Extra-pair copulations and mate guarding in the polyterritorial pied flycatcher, *Ficedula hypoleuca*. Behaviour.

[b3] Aljanabi SM, Martinez I (1997). Universal and rapid salt-extraction of high quality genomic DNA for PCR-based techniques. Nucleic Acids Res.

[b4] Andersson M (1994). Sexual selection.

[b5] Arnqvist G, Kirkpatrick M (2006). The evolution of infidelity in socially monogamous passerines: the strength of direct and indirect selection on extrapair copulation behavior in females. Am. Nat.

[b6] Birkhead TR, Møller AP (1998). Sperm competition and sexual selection.

[b7] Brommer JE, Alho JS, Biard C, Chapman JR, Charmantier A, Dreiss A, Hartley IR, Hjernquist MB, Kempenaers B, Komdeur J (2010). Passerine extra-pair mating dynamics: a Bayesian model comparison of four species. Am. Nat.

[b8] Coppack T, Tottrup AP, Spottiswoode C (2006). Degree of protandry reflects level of extrapair paternity in migratory songbirds. J. Ornithol.

[b9] Ellegren H (1992). Polymerase chain reaction (PCR) analysis of microsatellites: a new approach to studies of genetic relationships in birds. Auk.

[b10] Fisher RA (1930). The genetical theory of natural selection.

[b11] Garant D, Sheldon BC, Gustafsson L (2004). Climatic and temporal effects on the expression of secondary sexual characters: genetic and environmental components. Evolution.

[b12] Griffith SC, Owens IPF, Thuman KA (2002). Extra pair paternity in birds: a review of interspecific variation and adaptive function. Mol. Ecol.

[b13] Gustafsson L, Qvarnström A (2006). A test of the “sexy son” hypothesis: sons of polygenous collared flycatchers do not inherit their father's mating status. Am. Nat.

[b14] Gustafsson L, Qvarnström A, Sheldon BC (1995). A trade-off between a life-history and a secondary sexual trait. Nature.

[b15] Hjernquist MB (2008). Living in a variable environment.

[b16] Kalinowski ST, Taper ML, Marshall TC (2007). Revising how the computer program CERVUS accommodates genotyping error increases success in paternity assignment. Mol. Ecol.

[b17] Kirkpatrick M, Barton NH (1997). The strength of indirect selection on female mating preference. Proc. Natl. Acad. Sci. U. S. A.

[b18] Kokko H, Gunnarson TG, Morrell LJ (2006). Why do female migratory birds arrive later than males?. J. Anim. Ecol.

[b19] Leder EH, Karaiskou N, Primmer C (2008). Seventy new microsatellites for the pied flycatcher, *Ficedula hypoleuc*a and amplification in other passerine birds. Mol. Ecol. Res.

[b20] Lehtonen PK, Primmer CR, Laaksonen T (2009). Different traits affect gain of extrapair paternity and loss of paternity in the pied flycatcher, *Ficedula hypoleuca*. Anim. Behav.

[b21] Lundberg A, Alatalo R (1992). The pied flycatcher.

[b22] Merilä J, Sheldon BC (2000). Lifetime reproductive success and heritability in nature. Am. Nat.

[b23] Morbey YE, Ydenberg RC (2001). Protandrous arrival timing to breeding areas: a review. Ecol. Lett.

[b24] Price T, Kirkpatrick M, Arnold SJ (1988). Directional selection and the evolution of breeding date in birds. Science.

[b25] Qvarnström A (1997). Experimentally increased badge size increases male competition and reduces male parental care in the collared flycatcher. Proc. R. Soc. Lond. B.

[b26] Qvarnström A, Griffith SC, Gustafsson L (2000a). Male-male competition and parental care in collared flycatchers (*Ficedula albicollis*): an experiment controlling for differences in territory quality. Proc. R. Soc. Lond B.

[b27] Qvarnström A, Pärt T, Sheldon BC (2000b). Adaptive plasticity in mate preference linked to differences in reproductive effort. Nature.

[b28] Qvarnström A, Sheldon BC, Pärt T, Gustafsson L (2003). Male ornamentation, timing of breeding and the cost of polygyny in the collared flycatcher. Behav. Ecol.

[b29] Qvarnström A, Brommer JE, Gustafsson L (2006). Testing the genetic underlying the co-evolution of mate choice and ornament in the wild. Nature.

[b30] Rubolini D, Spina F, Saino N (2004). Protandry and sexual dimorphism in trans-Saharan migratory birds. Behav. Ecol.

[b31] Sheldon BC, Ellegren H (1999). Sexual selection resulting from extrapair paternity in collared flycatchers. Anim. Behav.

[b32] Sheldon BC, Merilä J, Qvarnström A, Gustafsson L, Ellegren H (1997). Paternal genetic contribution to offspring condition predicted by size of male secondary sexual character. Proc. R. Soc. Lond. B.

[b33] Sheldon BC, Andersson S, Griffith SC, Örnborg J, Sendecka J (1999). Ultraviolet colour variation influences blue tit sex ratios. Nature.

[b34] Török J, Hegyi G, Garamszegi LZ (2003). Depigmented wing patch size is a condition-dependent indicator of viability in male collared flycatchers. Behav. Ecol.

[b35] Veen, Träff J, Weissing FJ, Sheldon BC (2009). Reduced costs of mixed-species pairings in flycatchers: by-product or female strategy?. Behav. Ecol. Sociobiol.

[b36] Zahavi A (1975). Mate selection – selection for a handicap. J. Theor. Biol.

