# Supplementary material for: Experimental manipulation shows that the white wing patch in collared flycatchers is a male sexual ornament
Source: Ecol Evol. 2011 Dec;1(4):546–55. doi: 10.1002/ece3.48 (PMC3287330; doi:10.1002/ece3.48)
Supplement: Supplementary file 1 [file ece30001-0546-SD1.pdf]

## Supplementary information

Supplementary Table 1: Model results for the effect of treatment, area, date of capture and age (including interaction of age with treatment) on the probability to get a primary female. Age was considered in two categories: yearling (n = 28) or adult (n = 57). The contrast of the individual areas (in relation to the area 'Tuviken') are reported. Significant (P<0.05) variables are printed in bold.

| Term (Level) | coefficient | Wald $\chi^2$ | d.f. | P |
|---|---|---|---|---|
| *Probability to pair with a primary female* | | | | |
| **Intercept** | 2.34 ± 0.70 | 11.3 | 1 | <0.001 |
| Treatment (*Reduced*) | 0.56 ± 0.65 | 0.73 | 1 | 0.39 |
| **Area** | | 11.2 | 2 | 0.004 |
| *Anderse* | –1.58 ± 0.76 | | | |
| *Fide Prästäng* | –2.14 ± 0.65 | | | |
| **Date of capture** | –0.120 ± 0.042 | 8.1 | 1 | 0.004 |
| Age | 0.18 ± 0.74 | 0.06 | 1 | 0.81 |
| Age X Treatment | -1.74 ± 1.09 | 2.56 | 1 | 0.11 |

Fig. S1. Pictures of the wing patch prior (A) and after (B) experimental reduction,

Fig. S2. Pictures of the wing patch prior (A) and after (B) control painting.

*Pictures illustrating the reduction of the white wing patch by painting.*

As described in the main text, the wing patch size was reduced by blackening part of the white 'band' across primary, secondary and tertiaries that collared flycatchers display. The aim was to reduce this band to only a narrow band of white. The white on feathers was blackened from the tip-side of the feathers inwards(Fig. S1). Sham control painting (with a transparent marker) did not produce a noticeable difference (Fig. S2). All pictures by M de Heij.

For Review Only

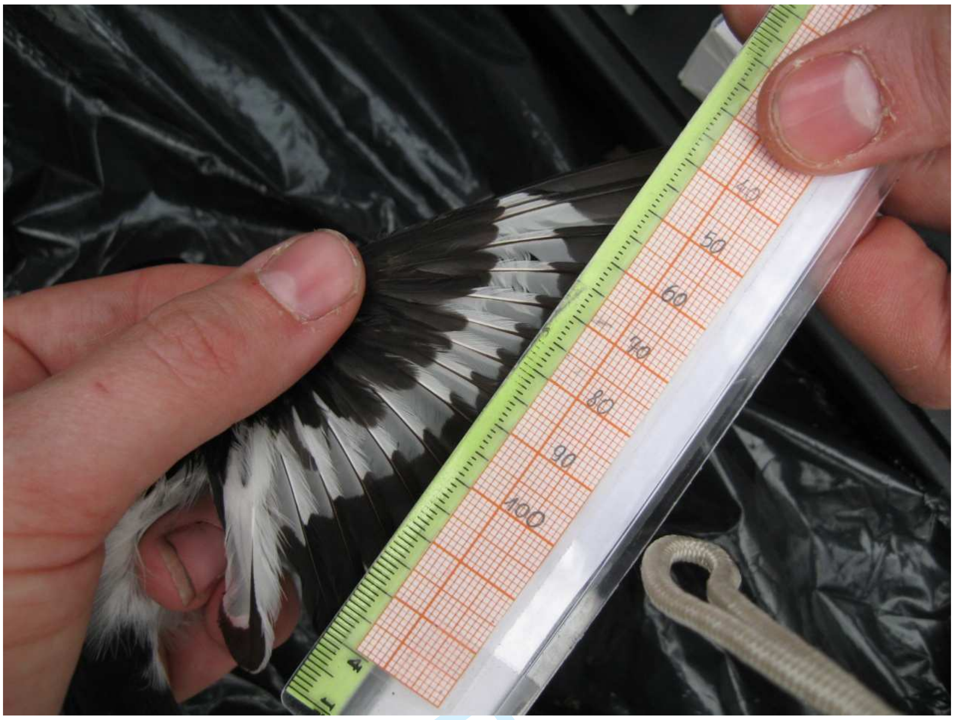

**Fig. S1 A**

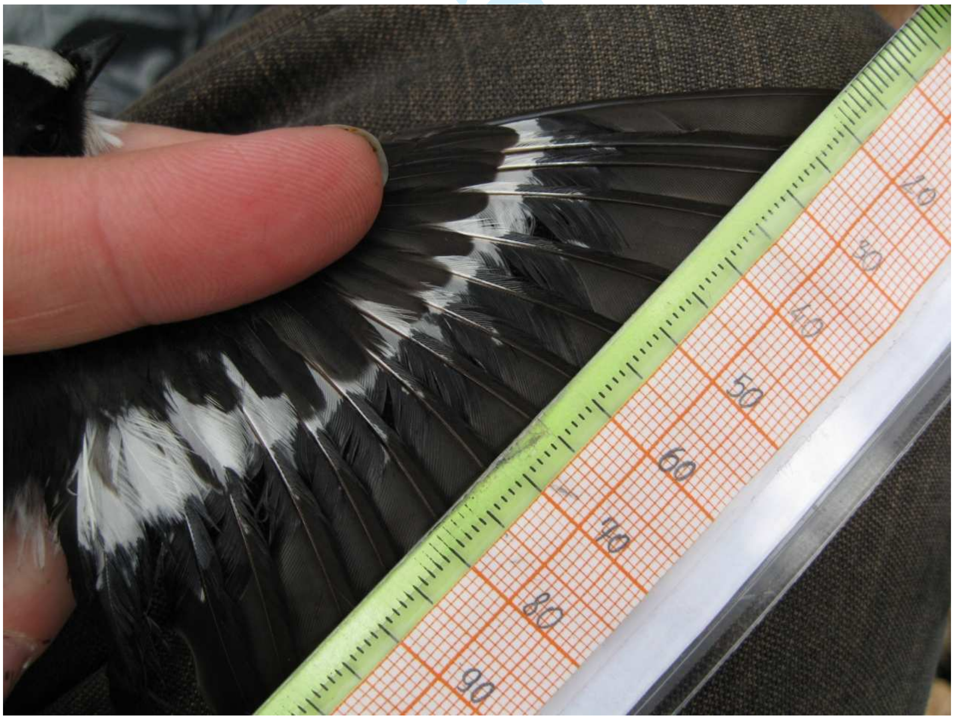

**Fig. S1 B**

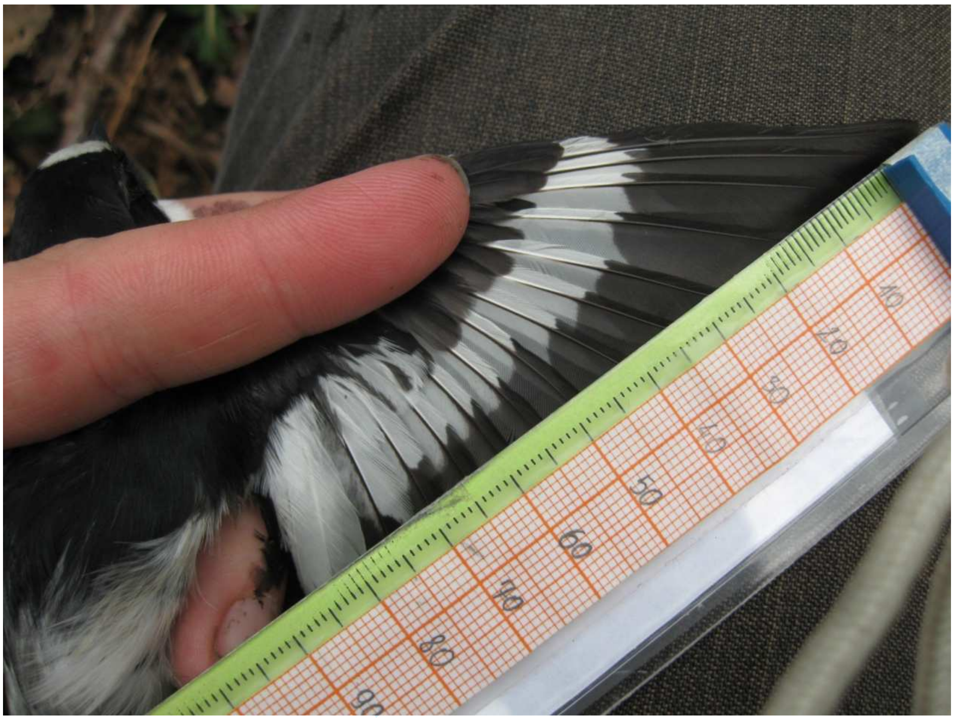

**Fig. S2 A**

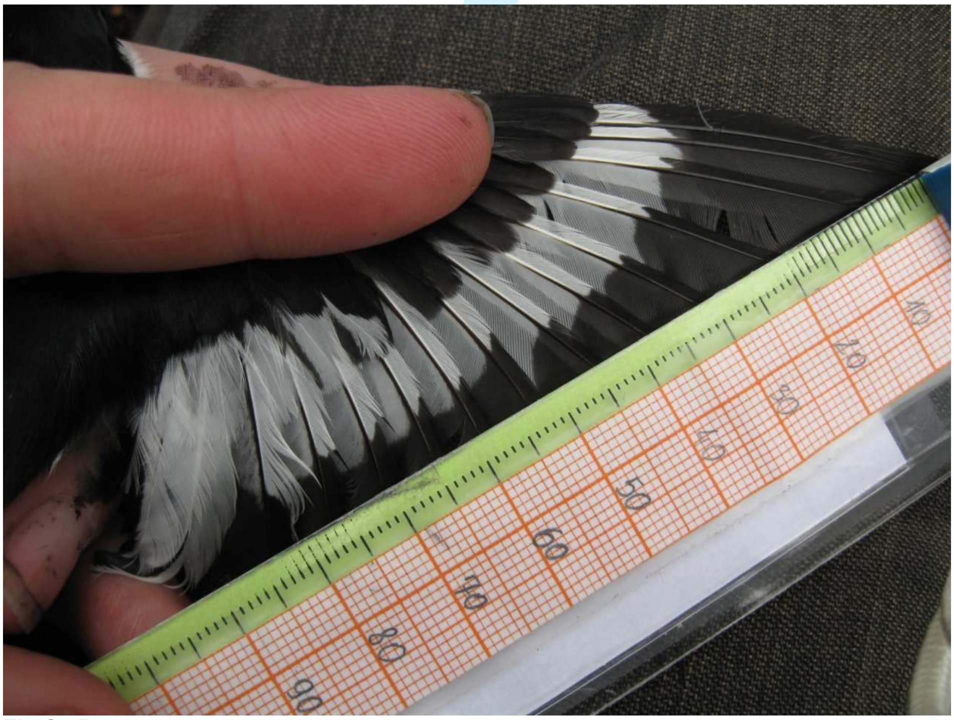

**Fig S2 B**
